# The association of umbilical hyperlactatemia with short- and tong-term outcomes in extremely low birth weight neonates: a matched cohort study

**DOI:** 10.1007/s00431-025-06147-z

**Published:** 2025-05-07

**Authors:** Leon Hirsch, Markus Waitz, Andreas Jenke

**Affiliations:** 1 University of Witten/Herdecke, Department of Pediatrics, Faculty of Health, Witten, Germany; 2 Children´s Hospital Kassel, Department of Neonatology and Paediatric Gastroenterology, Kassel, Germany

**Keywords:** ELBW, PH, Lactic acidosis, Lactate, Umbilical ABG, Bayley III

## Abstract

The objective of this study is to quantify the impact of umbilical arterial isolated hyperlactataemia and lactic acidosis on mortality, morbidity, and neurodevelopmental outcomes at 24 months corrected age in extremely low birth weight (ELBW) infants. The study population consisted of ELBW patients born between January 1, 2015, and December 31, 2023, at the Klinikum Kassel, receiving umbilical arterial blood analysis immediately after birth. Isolated hyperlactataemia was defined as a lactate concentration > 7.00 mmol/L, lactic acidosis as a pH < 7.10 and a lactate > 7.00 mmol/L, controls as pH ≥ 7.10 and lactate ≤ 7 mmol/L. Outcomes were analysed utilising a 1:2 case–control matched approach. Using the hospital data management system, 241 patients were eligible, 150 controls, 47 with isolated hyperlactataemia and 26 with lactic acidosis. Increased 28-day mortality was observed in both isolated hyperlactataemia patients (continuity corrected aOR 29.60 [1.63, 537.76], *p* = 0.022), and lactic acidosis patients (aOR 27.00 [3.18, 228.96], *p* = 0.003). Isolated hyperlactataemia was also associated with an increased risk for NEC stage ≥ 2 (aOR 10.00 [1.17, 85.59]), BPD ≥ moderate (aOR 2.68 [1.08, 6.65]), ROP grade ≥ 2 (aOR 13.77 [4.12, 46.02]), IVH grade ≥ 2 (aOR 2.91 [1.04, 8.13]), and septicaemia (aHR 16.76 [7.97, 35.22]). Lactic acidosis patients showed a more pronounced risk for adverse short-term outcomes (ROP grade ≥ 2 (aOR 29.33 [3.23, 266.23]), IVH grade ≥ 2 (aOR 6.31 [1.29, 30.74]), and septicaemia (aOR 1.97 [0.86, 4.54])). Neurodevelopmental outcomes did not differ between groups. *Conclusion*: Isolated hyperlactataemia and lactic acidosis significantly increased the risk of adverse short-term outcomes; however, neither had a significant effect on long-term developmental outcomes.

**Table:** 

**What is Known:** • *ELBW neonates face high risks of adverse outcomes, particularly with cord hyperlactatemia or lactic acidosis at birth. The long-term neurodevelopmental impact of these markers is unclear.*	
**What is New:** *• Lactic acidosis predicts worse short-term outcomes than hyperlactatemia. However, survivors to 24 months corrected age show comparable developmental outcomes.*

**Supplementary Information:**

The online version contains supplementary material available at 10.1007/s00431-025-06147-z.

## Introduction

Despite substantial advances in neonatal medicine, extremely low birth weight (ELBW, birth weight < 1000 g) and prematurity continue to be associated with increased risk of short- and long-term complications [1, 2]. This is reflected in a more than 20-fold increase in mortality and high incidence of serious morbidities as well as developmental deficits developed by one-third of ELBW survivors [1, 3, 4].

Accurate predictors of adverse outcomes are invaluable for clinical decision-making and parental counselling. Numerous prenatal factors—gestational age, birth weight, sex, multiple gestation, antenatal steroids, mode of delivery, etc.—have been explored and identified [5–8]. However, peri- and early postnatal factors have only recently moved into the focus of research with evidence accumulating that events in this time period have substantial impacts on short- and long-term outcomes [9–11]. Although asphyxia has been identified as one of the leading causes of neonatal mortality and morbidity in term infants, data on its identification and impact on outcomes in the ELBW population are limited [12].

Physiologically, asphyxia in neonatology is defined as a critical perinatal hypoxic state, with varying diagnostic definitions depending on country and guideline [13, 14]. The American Academy of Pediatrics and American College of Obstetrics and Gynecology defines neonatal asphyxia as the presence of all of the following: profound uABG (umbilical arterial blood gas) acidaemia (pH < 7.00), persistent Apgar scores ≤ 3 for longer than 5 min, neonatal neurologic sequelae and end organ dysfunction. These strict criteria, including neurological sequelae (e.g. hypotonia, abnormal oculomotor movements, weak or absent suck, apnoea, hyperpnea, or clinical seizures), are often based on definitions developed for term infants, but their application remains difficult in premature infants as they require extensive testing (i.e. EEG, USS or examination by a Paediatric Neurologist) [15].

The lack of a clear definition and specific diagnostic criteria for ELBW infants further limits its predictive value and impact on short- and long-term outcomes in this vulnerable population. To address this, clinical markers less affected by gestational age and birth weight yet available immediately postnatally (i.e. uABG markers such as pH and lactate) could help stratify the severity and guide therapeutic management of perinatal asphyxia. This hypothesis was supported by a recent study from the Canadian Neonatal Network on ELBW and extremely premature neonates, emphasising the importance of analysing and integrating metabolic markers to predict short-term outcomes [16]. Furthermore, a recent retrospective study revealed an increased risk of long term major developmental disability and IQ < 70 in preterm infants < 32 weeks gestational age with severe cord gas acidosis (pH < 7.0 and BE <  − 12) [17].

Acidosis is incorporated into many definitions of intrapartum hypoxia; however, arterial cord blood pH can be significantly affected by pCO_2_ levels, making it highly timing-dependent and limiting its ability to distinguish between hypoxemia and true tissue hypoxia. Whilst pH reflects overall acid–base status, it does not differentiate between respiratory and metabolic disturbances. In contrast, lactate is a direct marker of anaerobic metabolism and is therefore considered a more specific indicator of cellular hypoxia [18]. Importantly, a distinction must be made between isolated hyperlactataemia—elevated lactate in the absence of acidaemia—and lactic acidosis, which combines elevated lactate with reduced pH. The former may reflect early or recurrent transient, compensated hypoxic episodes, whereas the latter indicates more sustained or severe hypoxia with impaired metabolic compensation. The combined diagnostic or prognostic value of pH and lactate, compared to their individual use, remains unexplored in this patient population.

In this retrospective case–control matched cohort study, we aimed to quantify the effects of uABG hyperlactatemia in isolation and as lactic acidosis, on mortality, morbidity and long-term neurodevelopmental outcomes in ELBW infants.

## Methods

### Study design, setting and participants

A single-centre retrospective cohort study was conducted at the neonatal intensive care unit of the Klinikum Kassel (Gesundheit Nordhessen Holding AG Kassel, Germany). The study was conducted in line with the principles of the Declaration of Helsinki, reported adhering the EQUATOR Network STROBE guidelines, and ethics approval was granted by the Ethics Committee Witten/Herdecke (S- 123/2024).

Eligible were inborn ELBW infants who received an uABG immediately after delivery between January 1, 2015, and December 31, 2023, who were subsequently eligible for Bayley Scales of Infant and Toddler Development 3rd Edition (BSID-III) examinations. Noncord arterial, venous, or capillary BGA was not used due to its variable timing, thereby preventing the influence of postpartum clinical status and interventions on blood gas values. The exclusion criteria were outborn status, unknown time of birth or death (such as intrauterine death or death preceding uABG), noncord ABG, severe congenital malformations or syndromes unrelated to ELBW birth, and incomplete or missing data (including those who received intermittent or ongoing care at another hospital). Patients were identified using the Klinikum Kassel IQTIG (Institute for Quality Assurance and Transparency in Healthcare) reporting list. Identified ELBW neonates were categorised based on uABG pH and lactate. Isolated hyperlactataemia was defined as a pH ≥ 7.10 and a lactate concentration > 7.00 mmol/L, lactic acidosis as a pH < 7.10 and a lactate concentration > 7.00 mmol/L, and controls as pH ≥ 7.10 and a lactate concentration ≤ 7.00 mmol/L.

### Case–control matching

The nearest neighbour matching was performed using a one-case-to-two-control ratio, with blinding to outcomes by an independent statistician. This ratio was selected because of limitations in sample size. The recorded demographic and epidemiological variables were based on similar literature and on prenatal risk factors for hypoxia, asphyxia and increased short- and long-term morbidity [19]. The identified variables that could be analysed based on available data were antenatal corticosteroid administration, comorbidities of pregnancy, foetal transfusion syndrome, gestational age, gestational weight, intrauterine growth restriction, maternal age, multifetal pregnancy, noncephalic presentation, pathological Doppler, sex and mode of delivery. To avoid overmatching, a preliminary unmatched explorative statistical analysis was conducted with significant differences (*p* < 0.05) observed in antenatal corticosteroids, gestational age, gestational weight, sex, and pathological Doppler data. Matching was therefore based on these identified confounders, when more than two equal matches were identified per case, the controls born closest to each other chronologically were coupled, therefore receiving the most similar treatment.

### Definition of outcome variables, data sources, and sample size

Hyperlactatemia and lactic acidosis lack standardized definitions in ELBW neonates. Therefore, our cut-offs were informed by a 2023 meta-analysis by Matsushita, Krebs, and Carvalho, which analysed 62 studies on serum lactate and neonatal outcomes, identifying thresholds ranging from 2.5 to 9.95 mmol/L [20]. The average was pragmatically chosen in efforts to balance high sensitivity for adverse outcomes with broad inclusion of patients and, therefore, greater sensitivity.

The investigated outcomes were 28-day mortality, morbidities preceding death or discharge and long-term developmental outcomes. Morbidities included were patent ductus arteriosus (PDA) defined as haemodynamically significant at 28 days of life or time of death and diagnosed via transthoracic echocardiogram by an experienced paediatric cardiologist, necrotising enterocolitis stage ≥ 2 (NEC) defined according to the modified Bells criteria, bronchopulmonary dysplasia grade ≥ moderate (BPD) defined by Shennan 1988 and revised by the NICHD in 2016, retinopathy of prematurity grade ≥ 2 (ROP) defined by the International Classification of Retinopathy of Prematurity 3, intraventricular haemorrhage grade ≥ 2 (IVH) based on Papile’s classification modified for ultrasound, hyperbilirubinemia identified by phototherapy requirements and defined by the Maisels rule and nomograms ([gestational age—20] adjusted for the presence of risk factors) and definite neonatal sepsis (confirmed by positive blood cultures in the presence of clinical signs or raised inflammatory markers) [21–25].

Developmental outcomes were assessed at 24 months corrected age (calculated as chronological age minus the number of weeks premature) using BSID-III scores. Raw scores (noncomposite) were used to allow for fine- and gross-motor evaluation in isolation. As per the Bayley Scoring Manual, scores are on a scale of 1–19, with scoring categories of ≥ 7, < 7 to ≥ 4, < 4 to ≥ 1 and < 1, indicating normal outcomes, mild, moderate, and severe delays respectively.

Data were extracted from discharge summaries, ward round notes, lab reports, intensive care records, ventilation, medication, and prescription charts. Selection, measurement, and confounding bias were controlled for via a retrospective matched approach with blinding to outcomes. Blood gas values were based primarily on the analysis of the GEM5000 premier from Instrumentation Laboratory, employing potentiometric methods with ion-selective electrodes for precise hydrogen concentration measurements and amperometric detection via lactate oxidase for lactate concentrations [26, 27]. These methods result in a standard error of ± 0.01 pH units and ± 0.1 to ± 0.2 mmol/L of lactate [26].

The sample size was determined and constrained by the number of eligible ELBW patients born since the introduction of the BSID-III test program at Klinikum Kassel paediatric centre for chronic conditions and neonatal follow up clinic (Sozialpädiatrisches Zentrum) in 2016.

### Statistical analysis

Data collection and analysis were performed utilising descriptive statistics via Excel 2023, SPSS version 22 (SPSS 2016) and SAS Studio 3.81 (SAS Institute, USA, 2020). The paired nature of the data was considered, and symmetry was assessed using the Shapiro–Wilk test. Symmetrically distributed continuous outcomes were summarised using mean and standard deviation (SD) and further analysed using paired sample *t*-tests. Asymmetrically distributed continuous outcomes were summarised using median and interquartile ranges (IQR) and further analysed using the Wilcoxon signed-rank test. Categorical outcomes were summarised as number (percentage incidence), and differences were assessed using stratified multivariable logistic regression models employing the Wald Chi-Squared and Fisher’s scoring methods. Time-to-event data was assessed using Kaplan–Meier curves. All graphics were created using Microsoft Office 2023. The effect was assessed using adjusted odds (aOR) and hazard ratios (aHR) with 95% confidence intervals (CI), if the incidence in a case or control groups was 0, a continuity correction of 0.5 was used. *p*-values < 0.05 were considered significant.

## Results

**Fig. 1:**
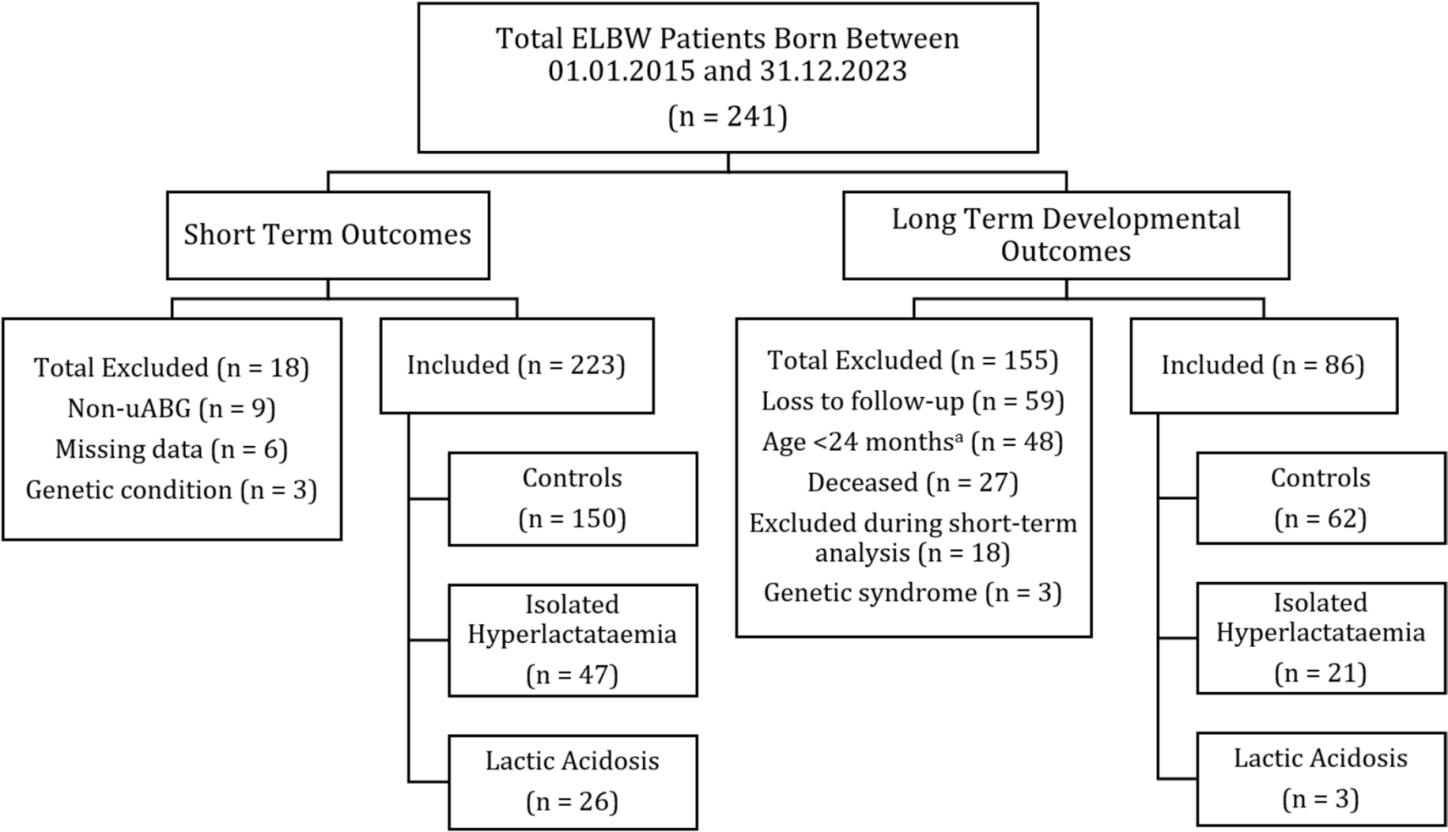
Flow diagram of ELBW (extremely low birth weight < 1000 g) patients included and excluded in short- and long-term outcomes. Isolated hyperlactatemia was defined as uABG (umbilical arterial blood gas) pH ≥ 7.10 and a lactate concentration > 7.00 mmol/L. Lactic acidosis was defined as uABG pH < 7.10 and a lactate > 7.00 mmol/L. Controls were defined as uABG pH ≥ 7.10 and a lactate ≤ 7.00 mmol/L. Age < 24 months was calculated using corrected age (chronological age minus the number of weeks premature)

Based on IQTIG reporting list, 241 patients were identified, 150 (62.2%) as controls, 47 (19.5%) with uABG isolated hyperlactataemia and 26 (10.8%) with lactic acidosis (Fig. 1). A total of 18 (7.5%) neonates were excluded, 9 (3.7%) due to delayed or noncord-BGA, 6 (2.5%) due to missing data, and 3 (1.2%) due to significant genetic or congenital abnormalities. At 24-month corrected age, 59 (24.4%) was lost to follow-up, 48 (19.9%) had not reached the 24-month corrected age, 27 (11.2%) deceased, 18 (7.5%) due to exclusion during short-term analysis, and 3 (1.2%) due to severe genetic or congenital neurodevelopmental conditions. Following the matching process, significant differences in baseline characteristics were observed only in independent variables of pH and lactate (Table 1). Nonsignificant differences were observed in all other demographic and epidemiological variables.

**Table 1:** Epidemiological and demographic characteristics of ELBW neonates by marker as part of a matched 1:2 case–control analysis

	Matched Isolated Hyperlactataemia Group			Matched Lactic Acidosis Group		
	Hyperlactataemia Control Group	Hyperlactataemia Group	MHG *P*-value	Lactic Acidosis Control Group	Lactic Acidosis Group	MLAG *P*-value
n	94 (39.0%)	47 (19.5%)		52 (21.6%)	26 (10.8%)	
pH values^a^	7.27 (± 0.1)	7.19 (± 0.1)	<.001	7.27 (± 0.1)	7.03 (± 0.1)	<.001
Lactate (mmol/L)^a^	3.73 (± 1.6)	9.61 (± 2.3)	<.001	3.26 (± 1.5)	13.14 (± 4.2)	<.001
Maternal age (years)^a^	30.11 (± 5.6)	31.13 (± 7.10)	.601	29.85 (± 6.0)	30.30 (± 6.2)	.704
Gestational weight (grams)^b^	730 (620–870)	740 (622.5–890)	.519	780 (515–940)	780 (495–952.5)	.764
Gestational age (weeks)^a^	26.65 (± 2.3)	26.47 (± 2.1)	.185	26.90 (± 2.2)	26.51 (± 2.3)	.638
Male sex^c^	33 (35.1%)	22 (46.8%)	.632	34 (65.4%)	21 (80.8%)	.274
Multiple gestation^c^	19 (20.2%)	14 (30.0%)	.659	14 (26.9%)	6 (19.2%)	.290
Single pregnancy^c^	75 (80.0%)	33 (70.2%)	.206	38 (73.1%)	20 (76.9%)	.714
Twin pregnancy^c^	16 (17.0%)	8 (17.0%)	1.000	11 (21.1%)	5 (19.2%)	.843
Triplets or Quadruplet pregnancy^c^	3 (3.2%)	6 (12.8%)	.060	3 (5.8%)	1 (3.8%)	1.000
Caesarean delivery^c^	89 (94.7%)	45 (95.7%)	.939	48 (92.3%)	25 (96.2%)	.416
Noncephalic presentation^c^	42 (44.7%)	23 (48.9%)	.168	21 (40.4%)	10 (38.5%)	.741
Antenatal corticosteroids^c,d^	57 (60.6%)	23 (48.9%)	.625	28 (52.8%)	9 (34.6%)	.069
Intrauterine growth restriction^c^	1 (1.1%)	2 (4.3%)	.096	2 (3.9%)	1 (3.9%)	.757
Pathological Doppler^c^	30 (31.9%)	11 (23.4%)	.053	19 (36.5%)	11 (42.3%)	.896
Co-morbidities of pregnancy^c,e^	36 (38.3%)	18 (38.3%)	.768	23 (44.2%)	10 (38.5%)	.946
Foetal transfusion syndrome (Donor)^c^	0 (0.0%)	2 (4.3%)	.994	0 (0.0%)	0 (0.0%)	1.000
Placental abruption or rupture^c^	4 (4.3%)	2 (4.3%)	1.000	5 (9.62%)	3 (11.5%)	.833

Data shown as ^a^mean (± standard deviation), ^b^median (Q1-Q3 [interquartile range]) and ^c^n (%). The groups were defined by umbilical arterial pH and lactate values, recorded immediately postnatal. The Matched Hyperlactatemia Group (MHG) was defined as those with a pH ≥ 7.10 and a lactate concentration > 7.00 mmol/L; the Lactic Acidosis Group (MLAG) was defined as pH < 7.10 and a lactate > 7.00 mmol/L; and the respectively matched Control Groups were defined as pH ≥ 7.10 and a lactate ≤ 7.00 mmol/L. Antenatal corticosteroids was defined as two doses of betamethasone, administered 24 h apart preceding birth. Co-morbidities of pregnancy were defined as a diagnosed HELLP, Pre-eclampsia, GDM, T1DM, or teratogenic substance consumption

### Impact of lactic acidosis and isolated hyperlactataemia on short term outcomes

**Fig. 2:**
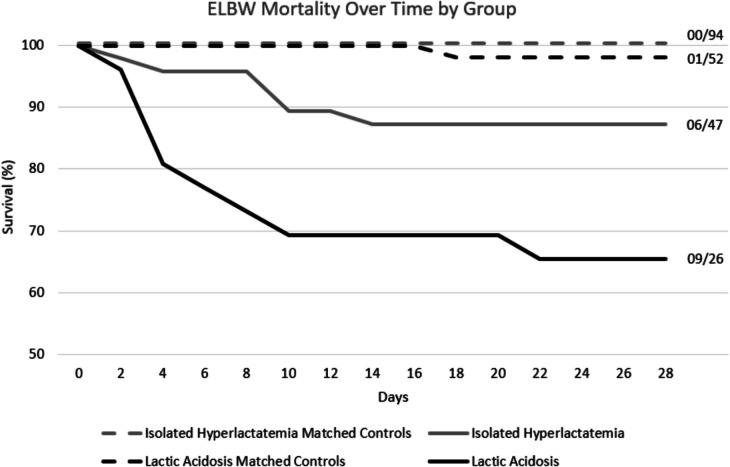
Inverse Kaplan–Meier curves of ELBW (extremely low birth weight < 1000 g) mortality until 28-days of life, matched 1:2 case–control. Isolated hyperlactatemia was defined as uABG (umbilical arterial blood gas) pH ≥ 7.10 and a lactate concentration > 7 mmol/L. Lactic acidosis was defined as uABG pH < 7.10 and a lactate > 7.00 mmol/L. Their respective controls were defined as uABG pH ≥ 7.10 and a lactate ≤ 7 mmol/L. Survival was displayed as percent survival on a scale of 50—100%, days survived was displayed in 2-day intervals

The time-to-event analysis of 28-day mortality revealed a 12.8% increased incidence in patients with isolated hyperlactataemia compared with controls (continuity corrected aOR 29.60 [95% CI: 1.63, 537.76], *p* = 0.022). In contrast, patients with lactic acidosis had a 32.7% increased incidence compared with their matched controls (aOR 27.00 [3.18, 288.96], *p* = 0.003) (Fig. 2).

Compared with controls, ELBW infants with isolated hyperlactataemia showed significantly greater odds for all measured morbidities (Table 2). The adjusted odds ratios were as follows: PDA (aOR 10.78 [3.72, 31.29], *p* < 0.001), NEC stage ≥ 2 (aOR 10.00 [1.17, 85.59], *p* = 0.036), BPD grade ≥ moderate (aOR 2.68 [1.08, 6.65], *p* = 0.033, adjusted for six patients deceased prior to grading (adj.), ROP grade ≥ 2 (aOR 13.77 [4.12, 46.02], *p* < 0.001, adj.6), IVH grade ≥ 2 (aOR 2.91 [1.04, 8.13], *p* = 0.042), and hyperbilirubinemia (aOR 4.84 [2.02, 11.55], *p* < 0.001, adj.2).

**Table 2:** Adjusted odds ratios of ELBW neonate morbidities, by morbidity and marker as part of the 1:2 case-controlled matched analysis

	MHG: Isolated Hyperlactatemia vs Control	*P*-value MHG	MLAG: Lactic Acidosis vs Control	*P*-value MLAG
n	47 vs 94		26 vs 52	
PDA	10.78 (3.72–31.29)	<.001	13.83 (3.15–60.67)	<.001
NEC stage ≥ 2	10.00 (1.17–85.59)	.036	7.20 (0.81–64.17)	.077
BPD grade ≥ moderate	2.68 (1.08–6.65)^a^	.033	3.14 (0.76–13.00)^b^	.115
ROP grade ≥ 2	13.77 (4.12–46.02)^a^	<.001	29.33 (3.23–266.23)^b^	.003
IVH grade ≥ 2	2.91 (1.04–8.13)	.042	6.31 (1.29–30.74)	.023
Hyperbilirubinemia	4.84 (2.02–11.55)^c^	<.001	16.36 (2.10–127.66)^d^	.008

Data is displayed as aOR (95% confidence intervals). The groups were defined by umbilical arterial pH and lactate values, recorded immediately postnatal. The Matched Hyperlactatemia Group (MHG) was defined as those with a pH ≥ 7.10 and a lactate concentration > 7.00 mmol/L; the Lactic Acidosis Group (MLAG) was defined as pH < 7.10 and a lactate > 7.00 mmol/L; and the respectively matched Control Groups were defined as pH ≥ 7.10 and a lactate ≤ 7.00 mmol/L. PDA was defined as haemodynamically significant patent ductus arteriosus at 28 days of life or time of death and diagnosed via transthoracic echocardiogram by an experienced paediatric cardiologist. Hyperbilirubinemia was identified by phototherapy requirements and defined by Maisels rule and nomograms ([gestational age—20] adjusted by presence of risk factors)

^b^^, c, e, f^ Adjusted for x patients deceased prior to testing. ^a^(6), ^b^(9), ^c^(2) and ^d^(5)

Similar results were seen for ELBW infants with lactic acidosis, displaying greater odds for all measured morbidities except NEC. The adjusted odds ratios were as follows: PDA (aOR 13.83 [3.15, 60.67], *p* < 0.001), NEC stage ≥ 2 (aOR 7.20 [0.81, 64.17], *p* = 0.077), BPD grade ≥ moderate (aOR 3.14 [0.76, 13.00], *p* = 0.115, adj.9), ROP grade ≥ 2 (aOR 29.33 [3.23, 266.23], *p* = 0.003, adj.9), IVH grade ≥ 2 (aOR 6.31 [1.29, 30.74], *p* = 0.023), and hyperbilirubinemia (aOR 16.36 [2.10, 127.66], *p* = 0.008, adj.5).

**Fig. 3:**
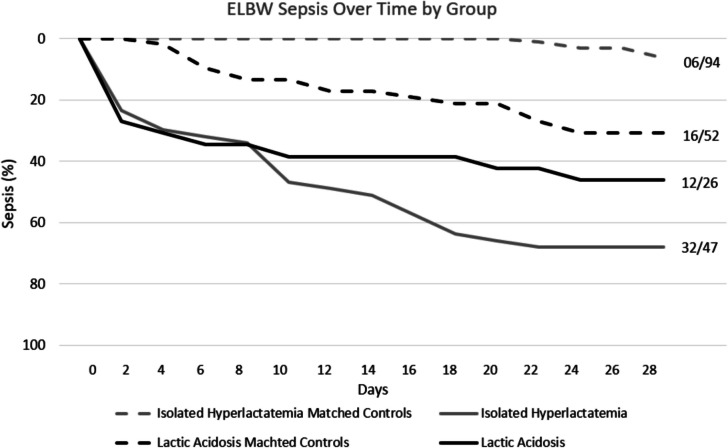
Inverse Kaplan–Meier curves of ELBW (extremely low birth weight < 1000 g) incidence of sepsis until 28-days of life, matched 1:2 case–control. Isolated hyperlactatemia (grey continuous line) was defined as uABG (umbilical arterial blood gas) pH ≥ 7.10 and a lactate concentration > 7.00 mmol/L. Lactic acidosis (black continuous line) was defined as uABG pH < 7.10 and a lactate > 7.00 mmol/L. Their respective controls (grey and black dashed lines respectively) were defined as uABG pH ≥ 7.10 and a lactate ≤ 7 mmol/L. Incidence was displayed as affected number/total number at the end of each line. The incidence of sepsis was presented as the percentage of surviving patients diagnosed on a scale of 0—100% in 2-day intervals

According to the time-to-event data analysis of sepsis (Fig. 3), infants with isolated hyperlactataemia presented a 61.7% increase in 28-day incidence of sepsis (aHR 16.76 [7.97, 35.22], *p* < 0.001). Patients with lactic acidosis showed a similar trend, with a 15.4% increase in incidence; however, the 95% CI and *p*-values (aHR 1.97 [0.86, 4.54], *p* = 0.088) were not significant.

### Effects of isolated hyperlactatemia and lactic acidosis on 24-month neurodevelopmental outcomes

In infants with isolated hyperlactataemia, follow-up examinations were performed on 21 patients (44.7%). The difference in multifactorial loss to follow up between case and controls was 4.3% (*p* = 0.321). The outcomes of the BSID-III examinations revealed nonsignificant differences in the cognitive (5.66 vs.7.50, *p* = 0.057), fine-motor (7.90 vs.8.90, *p* = 0.427), and gross-motor (6.76 vs.7.57, *p* = 0.365) domains. For infants with lactic acidosis, follow-up was performed on three patients (11.1%), with a significant difference between the case and control groups (48.1%, *p* = 0.010). Developmental outcomes were not significantly different: cognition (6.33 vs.7.50, *p* = 0.792), fine-motor (7.33 vs.9.33, *p* = 0.419), and gross-motor (6.67 vs.8.83, *p* = 0.694). Neither group showed an increased incidence of severe developmental impairment in comparison with their controls (*p* > 0.050).

### Lactate threshold exploration

The post hoc secondary analysis of short-term outcomes using a lactate threshold of 5 mmol/L followed the same methodology and dataset. Due to a limited sample size 1:1 matching was conducted for the 84 eligible patients with isolated hyperlactatemia (> 5.00 mmol/L), whilst the lactic acidosis group remained unchanged (*n* = 26), preserving matching and results (see supplemental figures). After matching, epidemiological and demographic variables showed no significant differences. Time-to-event analysis of mortality yielded an aOR of 2.84 (95% CI 0.72–11.11, *p* = 0.133). All short-term outcomes were nonsignificant (aOR overlapping 1, *p* > 0.05). However, time-to-event analysis of sepsis incidence was significant (aHR 2.13, 95% CI 1.85–2.45, *p* < 0.001).

## Discussion

This retrospective case–control cohort study is the first to specifically examine lactate levels in uABG samples in premature infants and their relationship to clinical outcomes. Our findings demonstrated that uABG hyperlactatemia, both in isolation and as lactic acidosis, was associated with a ≥ 27-fold increased risk of 28-day mortality and significantly elevated rates of PDA, ROP, IVH, hyperbilirubinemia, and sepsis in ELBW infants. These results align with previous studies assessing abnormal blood gas results but expand the scope by focusing on uABG, which provides direct insights into intrauterine hypoxia or ischemia.

Randolph et al. reported increased mortality in 3,979 ELBW infants with perinatal acidosis, defined as pH < 7.0 or BE <  − 12 [28]. Similarly, data from the Canadian Neonatal Network, including 2257 infants born between 23 + 0 and 28 + 6 weeks of gestation, demonstrated that lactate levels > 3 mmol/L were associated with a heightened probability of mortality [16].

A similar analysis of isolated hyperlactatemia was recently published using retrospective data from a German registry by Zipf and colleagues [29]. Their study investigated isolated hyperlactatemia in a population of 2499 infants born at < 29 weeks gestational age, measured < 24-h postnatal. Their findings linked a BGA lactate concentration exceeding 4 mmol/L with an increased risk of IVH and BPD, but not with sepsis or mortality. These contrasting results may be explained by their higher mean gestational age, birth weight and rate of antenatal steroid administration. Further, the direct comparison of outcomes between the two studies is impeded due to our exclusive inclusion uABG results, reflecting intrauterine hypoxia or ischemia only, whereas Zipf et al. excluded uABG, focusing on nonumbilical arterial, venous and capillary BGAs [29]. Elevated lactate levels (> 4 mmol/L) in their cohort were associated with an increased risk of IVH and BPD but not sepsis or mortality. The contrasting findings may be explained by differences in methodology, as Zipf et al. excluded uABG samples, reflecting a broader spectrum of hyperlactataemia causes such as postnatal sepsis and metabolic disturbances. Our exclusive analysis of uABG, by contrast, highlights the specific role of intrauterine hypoxic-ischemic events.

Pathophysiologically, elevated lactate levels disrupt cellular signalling pathways, induce inflammation, and exert immunosuppressive effects, exacerbating morbidity and mortality risks, especially in immature organ systems [30–32]. The higher cut-off values and combined markers in our study (lactate > 7.0 mmol/L, pH < 7.1) indicate more severe circulatory compromise, accounting for the greater mortality rates observed. This was further reaffirmed by a post hoc secondary analysis using a reduced lactate threshold of > 5.00 mmol/L. In isolation, hyperlactataemia demonstrated a significant association with sepsis (aHR 2.13, 95% CI 1.85–2.45, *p* < 0.001), however, nonsignificant increases in mortality, PDA, NEC, BPD, ROP, IVH and hyperbilirubinemia. In combination, lactic acidosis results remain unchanged. These findings highlight the role of hyperlactatemia at the right threshold or in combination with acidosis in immune dysfunction and its potential as an early marker of neonatal morbidity and mortality risk.

Regarding short-term outcomes, this study uniquely provides data on the impact of isolated hyperlactatemia and lactic acidosis across a broad range of neonatal morbidities. Whilst previous research explored these conditions independently, our combined analysis underscores their compounded effects and reflects the established pathophysiology of perinatal asphyxia, characterized by hypoxic-ischemic injury, cellular necrosis, and apoptosis [33]. These findings support a risk-based approach for early prophylactic interventions targeting ELBW infants with significant uABG hyperlactatemia or lactic acidosis.

Neurodevelopmental outcomes at 24-month corrected age revealed no statistically significant differences between groups, although a trend toward impaired cognitive outcomes in patients with isolated hyperlactatemia was noted. Survivorship bias, influenced by the high rates of loss to follow-up (exceeding 50% in the hyperlactatemia group and nearly 90% in the lactic acidosis group), likely explains this observation.

Our study aligns with and extends existing literature, emphasising the significant impact of uABG hyperlactatemia and lactic acidosis on neonatal morbidity, mortality, and developmental outcomes [16, 28]. By focusing exclusively on uABG, we provide novel insights into the pathophysiology of intrauterine compromise. Our findings are drawn from a diverse metropolitan population, enhancing generalizability, but the single-centre design, small sample size, and significant loss to follow-up limit the long-term outcome analysis and external applicability [34]. Additionally, the case–control design introduces the potential for confounding, as data on variables such as ethnicity, socioeconomic status, and maternal factors were not available [11, 19]. Moreover, temporality, survival bias, selection bias and variability in limitations of diagnostic tests/criteria may have further influenced results e.g. the specificity and sensitivity of BSID-III test [35].

In conclusion, obstetricians and neonatologists should be aware of the detrimental consequences of uABG hyperlactatemia and lactic acidosis in ELBW infants. These findings emphasise the importance of prenatal decision-making regarding delivery timing and postnatal strategies for risk stratification, surveillance, and prophylactic interventions for high-risk neonates.

Future research should adopt a prospective, multicentre approach incorporating multiple blood gas analyses during the first days of life to define precise lactate thresholds with optimal sensitivity and specificity for adverse outcomes. This would further advance our understanding of the prognostic significance of uABG hyperlactatemia and its implications for clinical practice.

## Supplementary Information

Below is the link to the electronic supplementary material.

Supplementary file1 (DOCX 124 KB)

## Data Availability

No datasets were generated or analysed during the current study.

## Abbreviations

aHR: Adjusted hazard ratio
aOR: Adjusted odds ratio
BE: Base excess
BGA: Blood gas analysis
BPD: Bronchopulmonary dysplasia
CI: Confidence intervals
ELBW: Extremely low birth weight
IQTIG: Institute for Quality Assurance and Transparency in Healthcare
IVH: Intraventricular haemorrhage
NEC: Necrotising enterocolitis
PDA: Patent ductus arteriosus
ROP: Retinopathy of prematurity
uABG: Umbilical arterial blood gas
