# Supplementary material for: The association of umbilical hyperlactatemia with short- and tong-term outcomes in extremely low birth weight neonates: a matched cohort study
Source: Eur J Pediatr. 2025 May 7;184(6):328. doi: 10.1007/s00431-025-06147-z (PMC12058829; doi:10.1007/s00431-025-06147-z)
Supplement: Supplementary file 1 — Supplementary file1 (DOCX 124 KB) [file 431_2025_6147_MOESM1_ESM.docx]

**Fig.1** Flow diagram of ELBW (extremely low birth weight <1000g) patients included and excluded in a secondary short-term outcome analysis. Isolated hyperlactatemia was defined as uABG (umbilical arterial blood gas) pH ≥7.10 and a lactate concentration >5.00 mmol/L. Lactic acidosis was defined as uABG pH <7.10 and a lactate >5.00 mmol/L. Controls were defined as uABG pH ≥7.10 and a lactate ≤5.00 mmol/L and matched with blinding to outcomes


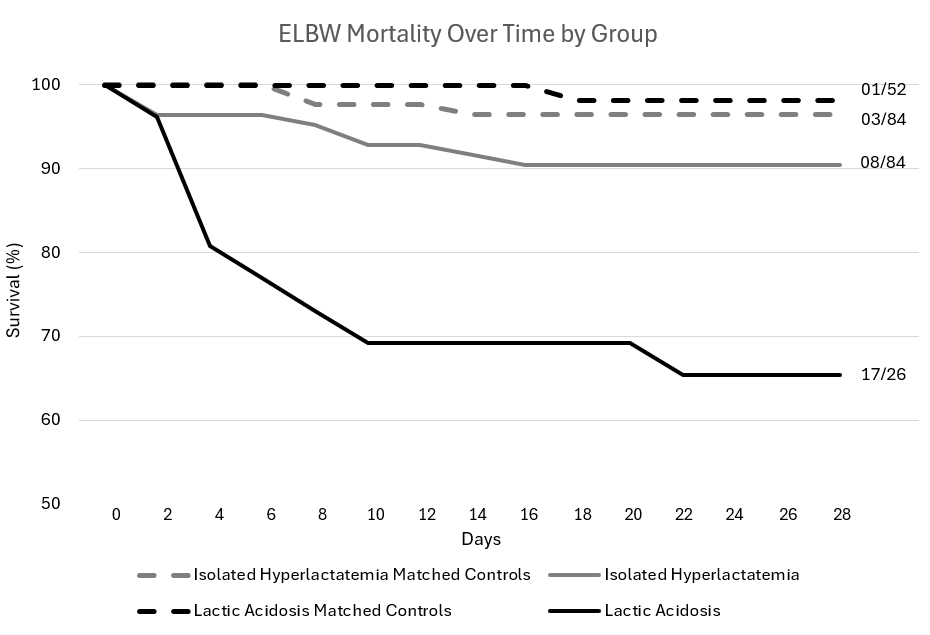


**Fig.2** Inverse Kaplan-Meier curves of ELBW (extremely low birth weight <1000g) mortality until 28-days of life. Isolated hyperlactatemia was defined as uABG (umbilical arterial blood gas) pH ≥7.10 and a lactate concentration >5mmol/L (1:1 matching due to sample size constraints). Lactic acidosis was defined as uABG pH <7.10 and a lactate >5.00 mmol/L. Their respective controls were defined as uABG pH ≥7.10 and a lactate ≤5mmol/L (2:1 matching). Survival was displayed as percent survival on a scale from 0 - 50%, days survived were displayed in 2-day intervals. Isolated Hyperlactataemia group aOR 2.84 (95% CI 0.72, 11.11) p=.133, Lactic Acidosis group aOR 29.60 (95%CI: 1.63, 537.76) p=.022


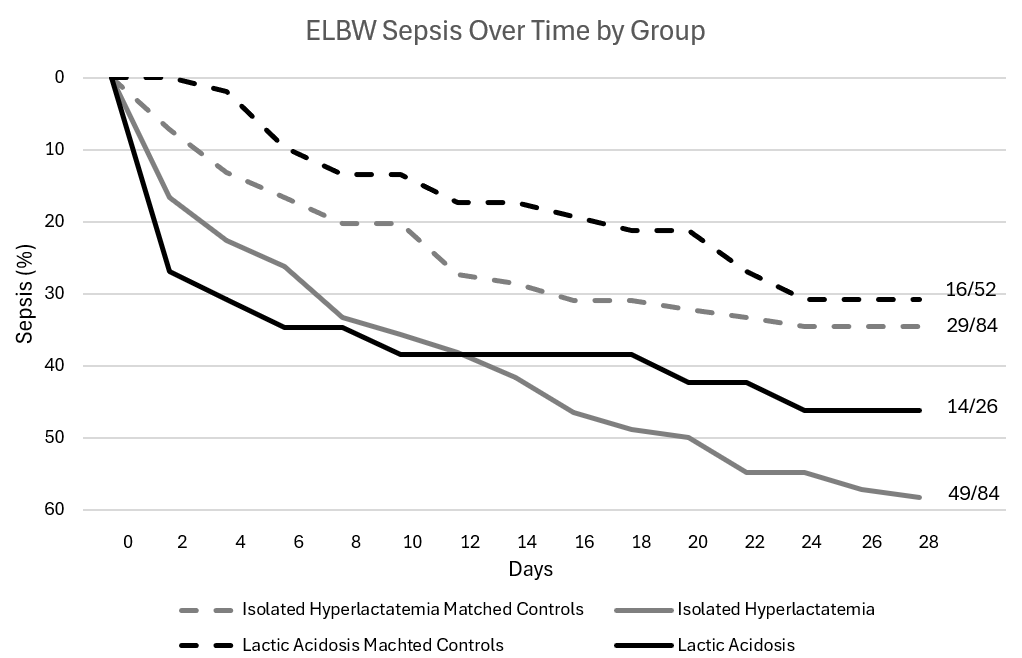


**Fig.3** Inverse Kaplan-Meier curves of ELBW (extremely low birth weight <1000g) incidence of sepsis until 28-days of life. Isolated hyperlactatemia was defined as uABG (umbilical arterial blood gas) pH ≥7.10 and a lactate concentration >5.00 mmol/L (1:1 matching due to sample size constraints). Lactic acidosis was defined as uABG pH <7.10 and a lactate >5.00 mmol/L (2:1 matching). Their respective controls were defined as uABG pH ≥7.10 and a lactate ≤5mmol/L. Incidence was displayed as affected number /total number at the end of each line. The incidence of sepsis was presented as the percentage of surviving patients diagnosed on a scale of 0 - 60% in 2-day intervals. Hyperlactataemia group aHR 2.13 (95%CI 1.85 to 2.45) p<.001, Lactic Acidosis group aHR 1.97 (0.86, 4.54) p=.088

**Table 1** Epidemiological and demographic characteristics of ELBW neonates by marker as part of a matched case-control analysis.

|  | Matched Isolated Hyperlactataemia Group | | | | Matched Lactic Acidosis Group | | |
| --- | --- | --- | --- | --- | --- | --- | --- |
|  | Matched Control Group | Hyperlactataemia Group | | MHG  P-value | Matched Control Group | Lactic Acidosis Group | MLAG  P-value |
| n | 84 | | 84 |  | 52 | 26 |  |
| pH values^a^ | 7.29 (±0.1) | | 7.19 (±0.1) | < .001 | 7.27 (±0.1) | 7.03 (±0.1) | <.001 |
| Lactate (mmol/L)^a^ | 2.65 (±1.0) | | 7.86 (±2.7) | < .001 | 3.26 (±1.5) | 13.14 (±4.2) | <.001 |
|  |  | |  |  |  |  |  |
| Maternal age (years)^a^ | 31.80 (±6.9) | | 30.80 (±6.5) | .478 | 29.85 (±6.0) | 30.30 (±6.2) | .704 |
| Gestational weight (grams)^b^ | 830 (677.5-970) | | 810 (670-902.5) | .142 | 780 (515-940) | 780 (495-952.5) | .764 |
| Gestational age (weeks)^a^ | 27.20 (±2.5) | | 26.70 (±2.1) | .230 | 26.90 (±2.2) | 26.51 (±2.3) | .638 |
| Male sex^c^ | 42 (50%) | | 44 (52.4%) | .758 | 34 (65.4%) | 21 (80.8%) | .274 |
|  |  | |  |  |  |  |  |
| Multiple gestation^c^ | 28 (33.3%) | | 24 (28.6%) | .589 | 14 (26.9%) | 6 (19.2%) | .290 |
| Single pregnancy^c^ | 56 (63,6%) | | 62 (70.4%) | .240 | 38 (73.1%) | 20 (76.9%) | .714 |
| Twin pregnancy^c^ | 21 (23.9%) | | 16 (18.2%) | .352 | 11 (21.1%) | 5 (19.2%) | .843 |
| Triplets or Quadruplet pregnancy^c^ | 7 (8.0%) | | 6 (6.8%) | 1.000 | 3 (5.8%) | 1 (3.8%) | 1.000 |
|  |  | |  |  |  |  |  |
| Caesarean delivery^c^ | 80 (95.2%) | | 79 (94.0%) | 1.000 | 48 (92.3%) | 25 (96.2%) | .416 |
| Non-cephalic presentation^c^ | 46 (45.2%) | | 37 (56.0%) | .165 | 21 (40.4%) | 10 (38.5%) | .741 |
| Antenatal  corticosteroids^c,d^ | 12 (14.3%) | | 21 (25.0%) | .081 | 28 (52.8%) | 9 (34.6%) | .069 |
| Intrauterine growth restriction^c^ | 3 (3.6%) | | 4 (4.8%) | 1.000 | 2 (3.9%) | 1 (3.9%) | .757 |
| Pathological Doppler^c^ | 21 (25%) | | 21 (25%) | 1.000 | 19 (36.5%) | 11 (42.3%) | .896 |
| Co-morbidities of pregnancy^c,e^ | 32 (38.1%) | | 32 (38.1%) | 1.000 | 23 (44.2%) | 10 (38.5%) | .946 |
| Foetal transfusion syndrome (Donor)^c^ | 1 (1,2%) | | 3 (3.6%) | 0.621 | 0 (0.0%) | 0 (0.0%) | 1.000 |
| Placental abruption or rupture^c^ | 7 (8.3%) | | 4 (4.8%) | .535 | 5 (9.62%) | 3 (11.5%) | .833 |

Data shown as ^a^mean (±standard deviation), ^b^median (Q1-Q3 [interquartile range]) and ^c^n (%). The groups were defined by umbilical arterial pH and lactate values, recorded immediately postnatal. The Matched Hyperlactatemia Group (MHG) was defined as those with a pH ≥7.10 and a lactate concentration >5.00 mmol/L (1:1 matching due to sample size constraints); the Lactic Acidosis Group (MLAG) was defined as pH <7.10 and a lactate >5.00 mmol/L (2:1 matching); the respectively matched Control Groups were defined as pH ≥7.10 and a lactate ≤5.00 mmol/L. Antenatal corticosteroids was defined as two doses of betamethasone, administered 24 hours apart preceding birth. Co-morbidities of pregnancy were defined as a diagnosed HELLP, Pre-eclampsia, GDM, T1DM, or teratogenic substance consumption

**Table 2** Adjusted odds ratios of ELBW neonate morbidities, by morbidity and marker as part of a case-controlled matched analysis.

|  | Isolated Hyperlactatemia Patient vs Matched Controls | P-value MHG | Lactic Acidosis Patients vs Matched Controls | P-value MLAG |
| --- | --- | --- | --- | --- |
| n | 84 vs 84 |  | 26 vs 52 |  |
| PDA | 1.26 (0.47 – 3.38) | .649 | 13.83 (3.15 – 60.67) | <.001 |
| NEC stage ≥ 2 | 6.60 (0.73 – 59.79) | .093 | 7.20 (0.81– 64.17) | .077 |
| BPD grade ≥ Moderate | 1.84 (0.80 – 4.25)^a^ | .151 | 3.14 (0.76 – 13.00)^b^ | .115 |
| ROP grade ≥ 2 | 2.07 (0.83 – 5.16)^a^ | .117 | 29.33 (3.23 – 266.23)^b^ | .003 |
| IVH grade ≥ 2 | 1.98 (0.54 – 7.29) | .306 | 6.31 (1.29 – 30.74) | .023 |
| Hyperbilirubinemia | 1.627 (0.748 – 3.54)^c^ | .219 | 16.36 (2.10 – 127.66)^d^ | .008 |

Data is displayed as aOR (95% confidence intervals). The groups were defined by umbilical arterial pH and lactate values, recorded immediately postnatal. Matched Hyperlactatemia Group (MHG) was defined as those with a pH ≥7.10 and a lactate concentration >5.00 mmol/L (1:1 matching due to sample size constraints); the Lactic Acidosis Group (MLAG) was defined as pH <7.10 and a lactate >5.00 mmol/L (2:1 matching); the respectively matched Control Groups were defined as pH ≥7.10 and a lactate ≤5.00 mmol/L. PDA was defined as haemodynamically significant patent ductus arteriosus at 28 days of life or time of death and diagnosed via transthoracic echocardiogram by an experienced paediatric cardiologist. Hyperbilirubinemia was identified by phototherapy requirements and defined by Maisels rule and nomograms ([gestational age - 20] adjusted by presence of risk factors)

^b, c, e, f^ Adjusted for x patients deceased prior to testing. ^a^(8), ^b^(9), ^c^(2) and ^d^(5)
